# Purification and Functional Characterization of a Protein: *Bombyx mori* Human Growth Hormone Like Protein in Silkworm Pupa

**DOI:** 10.1371/journal.pone.0114351

**Published:** 2014-12-03

**Authors:** Jianqing Chen, Tejun Shu, Zhengbing Lv, Zuoming Nie, Jian Chen, Hao Chen, Wei Yu, Qijing Gai, Yaozhou Zhang

**Affiliations:** 1 Zhejiang Provincial Key Laboratory of Silkworm Bioreactor and Biomedicine, College of Life Science, Zhejiang Sci-Tech University, Hangzhou 310018, China; 2 College of Pharmacy, Tianjin University, Tianjin 300073, China; Uppsala University, Sweden

## Abstract

Human growth hormone (hGH) is a peptide hormone secreted by eosinophils of the human anterior pituitary, and a regulatory factor for a variety of metabolic pathways. A 30-kD protein from the pupa stage of silkworm was detected by Western blotting and confirmed by immunoprecipitation based on its ability to bind to anti-hGH antibody. This protein, named BmhGH-like protein, was purified from fresh silkworm pupas through low-temperature homogenization, filtration, and centrifugation to remove large impurity particles. The supernatants were precipitated, resuspended, and passed through a molecular sieve. Further purification by affinity chromatography and two-dimensional electrophoresis resulted in pure protein for analysis by MS MALDI-TOF-MS analysis. An alignment with predicted proteins indicated that BmhGH-like protein consisted of two lipoproteins, which we named hGH-L1 and hGH-L2. These proteins belong to the β-trefoil superfamily, with β domains similar to the spatial structure of hGH. Assays with K562 cells demonstrated that these proteins could promote cell division *in vitro*. To further validate the growth-promoting effects, *hGH-L2* was cloned from pupa cDNA to create recombinant silkworm baculovirus vBmNPV-hGH-L2, which was used to infect silkworm BmN cells at low titer. Flow cytometric analysis demonstrated that the protein shortened the G0/G1 phase of the cells, and enabled the cells to rapidly traverse the G1/S phase transition point to enter S phase and promote cell division. Discovery of hGH-like protein in silkworm will once again arouse people’s interest in the potential medicinal value of silkworm and establish the basis for the development of new hormone drugs.

## Introduction

Human growth hormone (hGH) is a 191-amino-acid peptide hormone secreted by cells of the human anterior pituitary. It is a nonglycosylated hydrophilic single chain globulin with a relative molecular weight of approximately 22 kDa [1]. It has extensive and important physiological functions, including promotion of growth and development of most tissues (except nervous tissue), organs, and the whole body; regulation of the human body’s metabolism; and various other physiological functions. It is a primary postnatal growth-promoting hormone as well as an important regulatory factor of metabolic pathways. The hGH can affect the proliferation and differentiation of nearly all tissues and organs including bone tissue, cartilage tissue, adipose tissue, brain tissue, the immune system, the reproductive system, and the hematopoietic system [2]. It can regulate various metabolic processes and can be used in the treatment of certain diseases. However, during treatment, the body will produce antibodies against the hormone, leading to harmful side effects. Because of this, hormonal therapy has long been controversial. Therefore, the exploration of hormone analogues to reduce these side effects is currently one of the hotspots of investigation of new hormone-like protein drugs.

Growth hormone-like proteins have been identified in other organisms [3], [4]. Kaplan et al extracted a type of hGH-like protein from sheep’s testicles in 1967 [5]. Lechan et al detected hGH-like proteins in the rat brain using immunohistochemical methods in 1981 [6]. Swinnen et al reported the presence of hGH-like antigenic determinants in *Locusta migratoria* and *Sarcophaga bullata* neurons in 1990 [7]. Ominato et al identified an hGH-like protein in the pituitary of *Petromyzon marinus* in 2002 [8]. Gallardo et al found an hGH-like protein in rotifer using antibodies to the growth hormone of salmon, and demonstrated its function in promotion of rotifer reproduction [9]. Phares’s research group conducted a study on *Spirometra mansonoides* and found that the parasite secreted a protein that can bind to the hGH receptor [10].

For the first time, we detected an immunoreactive protein in silkworm chrysalis and haemolymph that reacts with an hGH antibody. The protein, which we named BmhGH-like protein, has a molecular weight of 30 kDa and is present as a monomer in its natural state [11]. The proteins were purified by molecular sieve with affinity chromatography using the characteristics of the binding of BmhGH-like protein to hGH antibody in the natural state. The purified proteins were separated using two-dimensional electrophoresis, processed by in-gel digestion, and then identified using MALDI-TOF mass spectrometry (MALDI-TOF-MS) analysis. The protein structure, biological function, and the relationship to hGH were further investigated to lay a foundation for exploring its potential as a new hormone protein drug.

## Materials and Methods

### 1 Main materials

The pupa and silkworm cell line BmN, originally purchased from Invitrogen, have been propagated and stored in our laboratory, human erythroleukemia K562 cells were a gift from the Institute of Hematology of Chinese Academy of Medical Sciences [12], standard hGH was purchased from Shanghai United Cell Biotechnology Co., Ltd., Plus Immobilization Kit was purchased from Thermo Corporation, mouse anti-hGH monoclonal antibodies were purchased from R & D Company, two-dimensional electrophoresis reagents and Superdex 200 prepacked columns were purchased from GE Healthcare, serum-free medium was purchased from Gibco, fetal bovine serum and RPMI1640 medium were purchased from Invitrogen, horseradish peroxidase (HRP)-conjugated goat.

### 2 Methods

#### 2.1 Pre-fractionation of the components of pupa

Fresh pupas were mixed with ice-cold phosphate buffer solutions at a certain proportion, homogenized three times at 4°C, and filtered through gauze to remove large impurities. The filtrates were centrifuged at 4°C and 15,000 rpm for 20 min, the pellets were discarded and supernatants were centrifuged again. The process was repeated three times, after which the final supernatants were collected. The proteins in the supernatants were precipitated with 60% ammonium sulfate overnight. The precipitated products were centrifuged at 4°C and 8000 rpm for 20 min, the supernatants were discarded, the pellets were re-suspended in cold PBS to re-dissolve the proteins, the solutions were filtered through 0.45 µm membranes, and depigmentation of the samples was performed using a Superdex 200 molecule sieve in the AKTA protein purification system. OD280 of the samples were then measured and absorption peaks of various proteins were collected and concentrated using a 10 kDa ultrafiltration tube. The proteins were quantitated with the BCA assay and separated by SDS-PAGE.

#### 2.2 Detection of BmhGH-like protein distribution using Western blot and Dot blot

After SDS-PAGE, the separated proteins were transferred to film at a constant voltage of 80 for 1.5 h, and the film was then blocked with 5% BSA for 2 h. The film was incubated in diluted primary antibody at 37°C for 2 h and rinsed with TBST (with 0.5% Tween-20) three times for 10 min each time. The film was then incubated in diluted secondary antibody at 37°C for 1 h, rinsed with TBST three times, and developed with DAB. Additionally, samples of the absorption peaks of each protein sample were transferred to a cellulose acetate membrane by vacuum filtration for Dot blot detection. The primary antibody was mouse anti-hGH monoclonal antibody, and the secondary antibody was horseradish peroxidase-labeled goat anti-mouse IgG (H + L).

#### 2.3 Purification of BmhGH-like protein by affinity chromatography

According to the Plus Immobilization Kit Operating Manual of Thermo Corporation, the affinity chromatography column was prepared by coupling 1.6 mg hGH monoclonal antibody [13]. The column was equilibrated at room temperature, and the protective solution was discarded. The column was then equilibrated again with five times its volume of Binding Buffer (0.15 M PBS, pH 7.4). The samples were filtered with a 0.45 µm membrane and loaded into the column, which was then incubated at room temperature on a shaker for 1 h. The permeabilizing solution was then discarded, and the column was washed with five times its volume of Wash Buffer (0.15 M PBS, pH 7.4) to remove the unbound molecules. Finally, multiple column volumes of Elution Buffer (0.1 M Glycine-HCl, pH 2.5) were added to elute the bound target proteins. The eluant was then adjusted to neutral pH using Neutralization Buffer (0.5 M Tris-HCl, pH 8.5). The protein in each sample was then quantitated by BCA assays and separated by SDS-PAGE.

#### 2.4 Two-dimensional electrophoresis and mass spectrometric identification of purified products

As outlined in the manual for GE Healthcare two-dimensional electrophoresis, 15 µL purified sample after BCA quantitation was thoroughly mixed with 75 µL Sample Lysis Buffer and 90 µL Rehydration Buffer, and the mixture was added into a 7 cm standard strip holder to ensure even distribution of the sample. The immobilized pH gradient (IPG) strip was then placed on the sample, 1 mL covering oil was added, and the first dimension isoelectric focusing was begun. After the focusing, the stripe was removed and placed into equilibration buffer containing 1% DTT and 2.5% iodoacetamide (75 mM Tris-HCl pH 8.8, 6 M Urea, 29.3% glycerol, 2% SDS, 0.002% bromophenol blue), and then equilibrated on an oscillator for 15 min. The equilibrated strip was then rinsed twice with double-distilled water and transferred to the surface of a prepared SDS-PAGE gel along with a filter paper containing a solution of standard molecular weight proteins. The strip was then sealed with 0.5% low melting point agarose. The second dimension electrophoresis was started at 10 mV for 15 min and continued at 20 mA for 5 h. Coomassie blue staining of the gel was performed after electrophoresis.

The protein spots were removed from the two-dimensional electrophoresis gel, placed into a new Eppendorf tube, 500 µL destaining solution (100 M NH_4_HCO_3_, 50% ACN) was added at room temperature for 1 h, and the liquid fraction was discarded. This process was then repeated. 100 µL 100% CAN was added to dehydrate the gel at room temperature for 5 min. The liquid fraction was removed, the tube was placed under vacuum drying for 30 min to remove ACN, and 20 µL digestion buffer with trypsin (40 M NH_4_HCO_3_, 10% CAN, 20 ug/mL trypsin) was added. The mixtures were left to stand at 4°C for 30 min and then at 37°C digested overnight. A tip containing C18 was used to pipet the protein, which was then dissolved in protein extract solution (α-cyano-4-hydroxycinnamic acid saturated solution with 50% ACN and 1% TFA).

The sample (1 µL) was used for spotting. The peptide mass fingerprint of the protein was obtained, and GPS Explorer V3.5 and Mascot were used to search the NCBI database to obtain the amino acid sequence of the protein and related information.

#### 2.5 BmhGH-like protein homology analysis and structure prediction

Multiple sequence alignment and homology analysis of the identified BmhGH-like protein were performed using growth hormone protein sequences from a variety of species (NCBI) using BioEdit software. Tertiary structure prediction was performed with a homology modeling method using phyre2 (http://www.sbg.bio.ic.ac.uk/phyre2).

#### 2.6 Biological functions of BmhGH-like protein

In vitro measurement of the growth-promoting effect of BmhGH-like protein was carried out in this study. The human erythroleukemia K562 cell culture system is one of the methods for in vitro measurement of biological activity of hGH. In serum-free culture medium, hGH at nanogram levels can promote cloning of K562 cells [14]. The cell suspension was diluted with RPMI-1640 medium and plated at a density of approximately 100 cells per well. Purified hGH-L was added, and an hGH standard was used as a positive control. The cells were cultured for four days and then observed under an inverted microscope. Any cell cluster containing four or more cells was counted as a clone. Clones were counted, and the numbers of clones of each concentration group were averaged. The relative colony-forming efficiency was calculated using the following formula: (Number of cell clones in the experimental group/Number of cell clones in negative control group)×100%, thereby determining the colony-forming promotion ability of BmhGH-like protein.

Based on the results of mass spectrometry analysis, NCBI was searched for the gene sequences encoding BmhGH-like protein. The target gene was then amplified using pupa cDNA as a template, and the amplified products were cloned into the transfer vector pFastBac1-ph, which was then transfected into DH10Bac competent cells. The cells were spread on a plate, and positive clones were selected to obtain the Bacmid recombinant virus plasmids. The plasmids were then transfected into silkworm BmN cells with lipofectamine, and recombinant virus containing the BmhGH-like gene were harvested. Silkworm BmN cells were infected with a low titer of virus; cells infected with wild virus were used as a control group. The cells were sampled at different times and flow cytometry was used to evaluate FSC, SSC, W, A and other parameters. The data were processed using FlowJo software, and the effects of BmhGH-like protein on the cell cycle were analyzed.

## Results and Analysis

### 1 Pre-fractionation and identification of BmhGH-like protein in silkworm pupas

**Figure 1:**
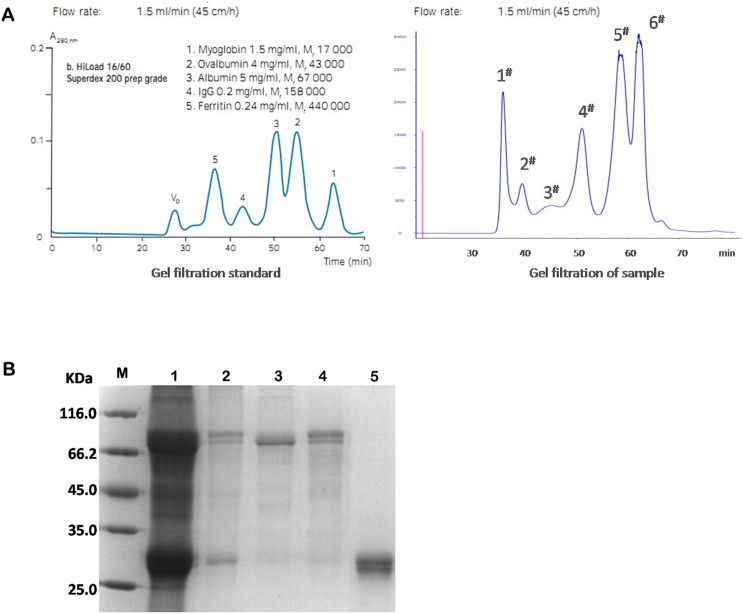
Preliminary isolation of BmhGH-like protein in silkworm pupa. A. Molecular sieve filtration of BmhGH-like proteins after ammonium sulfate precipitation. Right, Gel filtration standard. Left, Gel filtration of sample. B. SDS-PAGE analysis of various protein components filtered by molecular sieve. M. LMW protein marker; 1: total protein; 2: absorption peak #1; 3: absorption peak #2; 4: absorption peak #3; 5. absorption peak #4.

When the defatted protein samples were passed through the molecular sieve Superdex 200, six absorption peaks were collected, and the depigmentation effect was obvious. Based on the reference peaks and the color of the samples, absorption peaks 5 and 6 were presumed to be small molecules and pigments below 17 kDa: the sample of absorption peak 4 had a molecular weight of 17 kDa–43 kDa. Absorption peaks 1 and 4 were taken for separation by SDS-PAGE. As shown in Figure 1, good separation was achieved after passing the pupa samples through the molecular sieve, and the proteins collected in absorption peak 4 were approximately 30 kDa.

**Figure 2:**
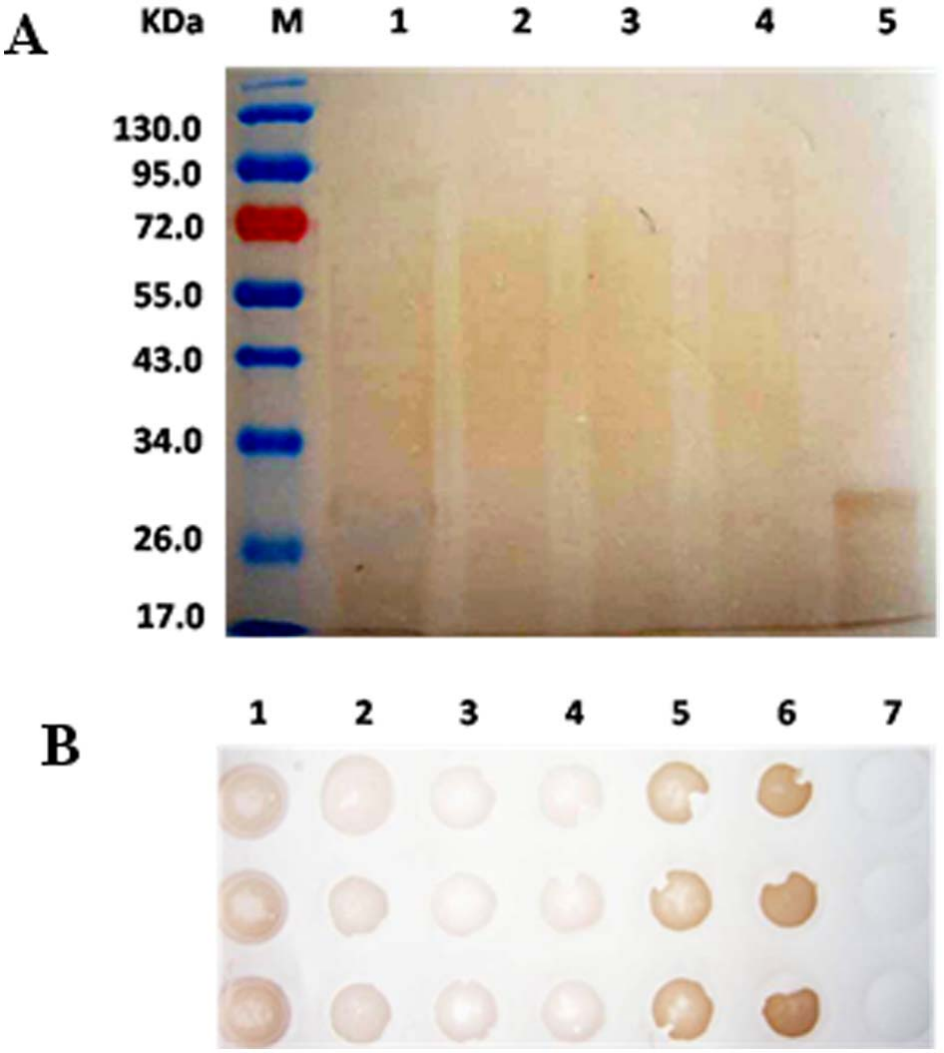
Identification of BmhGH-like protein by Western blot and Dot blot. Western blot. M: LMW protein marker; 1: total protein sample; 2: absorption peak #1; 3: absorption peak #2; 4: absorption peak #3; 5: absorption peak #4. B. Dot blot. 1: total protein sample; 2: absorption peak #1; 3: absorption peak #2; 4: absorption peak #3; 5: absorption peak #4; 6: hGH standard; 7: negative control.

The separated proteins were evaluated with the hGH antibody using Western blot and Dot blot tests. As shown in Figure 2, the BmhGH-like protein was clearly present in both the total protein sample and the sample of absorption peak 4. Because the pigment could not be completely removed, some background signal was present. However, the results showed that BmhGH-like protein could bind to hGH antibody in both denatured and natural states. Therefore, in subsequent experiments, antibody-coupled affinity chromatography was used to concentrate and isolate BmhGH-like protein from the protein sample of absorption peak 4.

### 2 Purification of BmhGH-like protein by affinity chromatography

**Figure 3:**
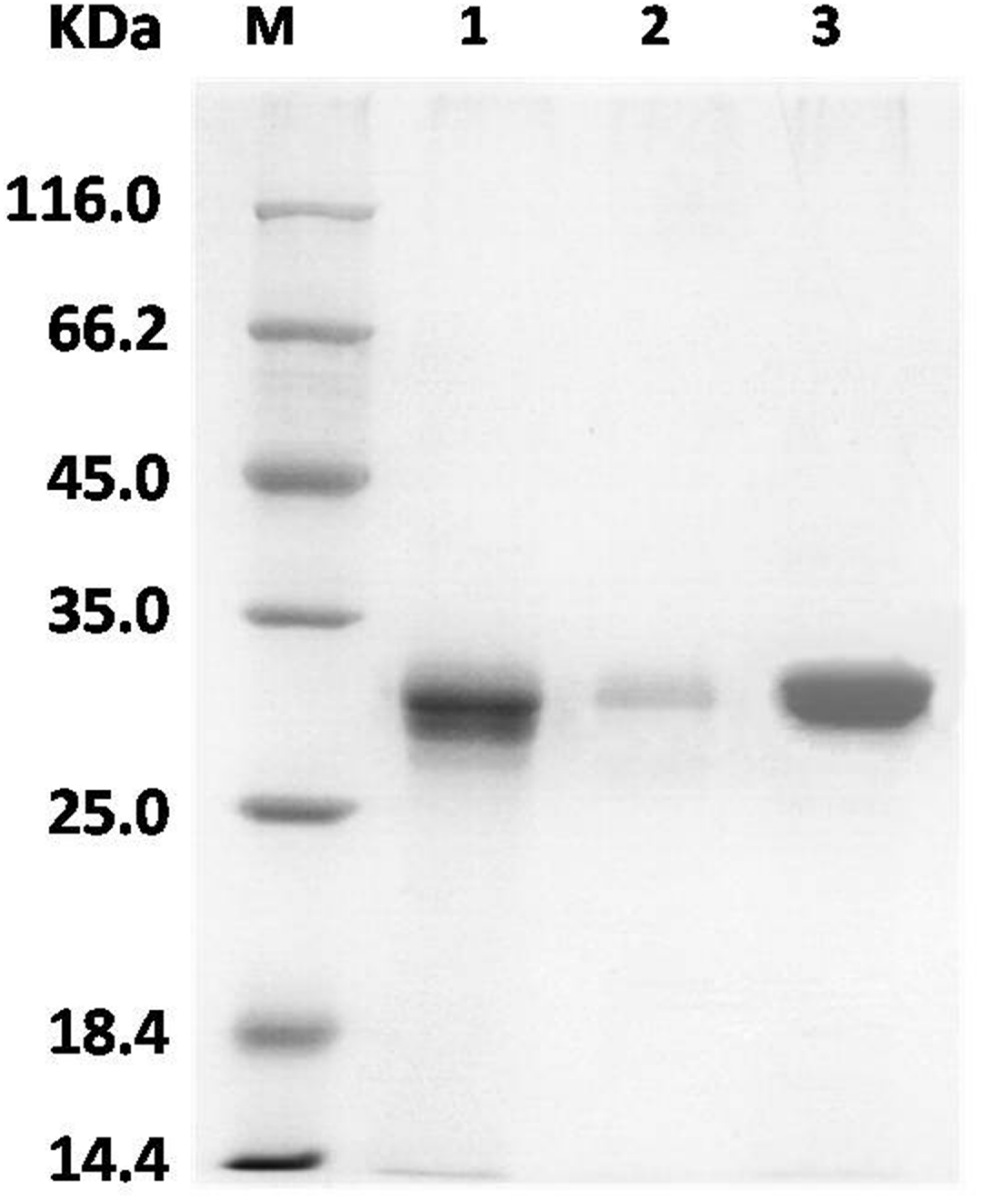
Purification of BmhGH-like protein by affinity chromatography. M: low molecular weight protein marker; 1: absorption peak #4; 2: eluted sample; 3: concentrated sample.

The hGH monoclonal antibody was coupled to the affinity chromatography column by its amino group through a chemical reaction. BmhGH-like protein was then captured on this column from the protein sample of absorption peak 4. As shown in Figure 3, the SDS-PAGE results indicated that affinity purification achieved the separation of the BmhGH-like protein from other components with high purity.

### 3 Two-dimensional electrophoresis and mass spectrometry identification of BmhGH-like protein

**Figure 4:**
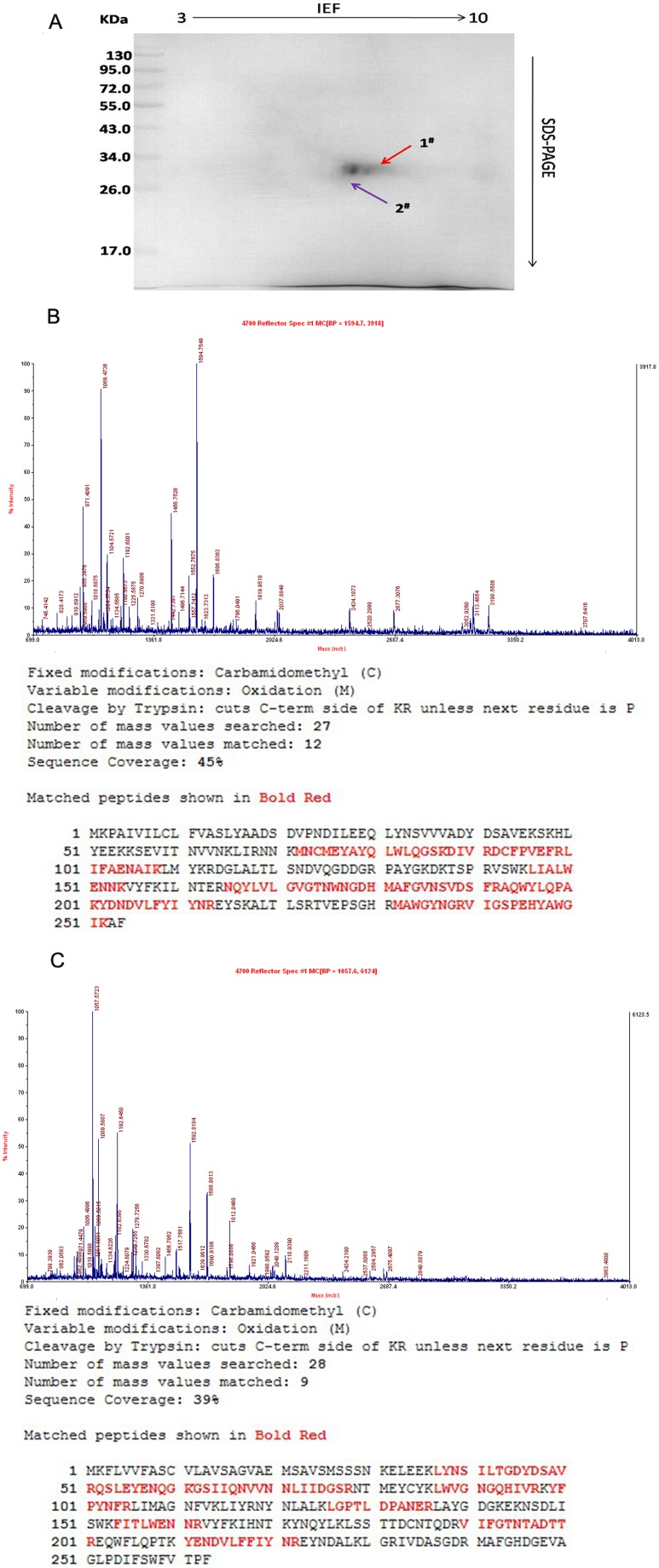
Identification of BmhGH-like protein by two-dimensional electrophoresis and mass spectroscopy. A. Two-dimensional electrophoresis assay. B. hGH-L1 peptide mass fingerprinting and protein match details. C. hGH-L2 peptide protein match details.

The purified proteins were further analyzed using two-dimensional electrophoresis. As shown in Figure 4a, the results suggested that the molecules binding to hGH antibodies were composed of two proteins with a molecular weight of approximately 30 kDa and very similar isoelectric points. The proteins were named hGH-L1 and hGH-L2.

After enzymolysis, the two proteins were evaluated by MALDI-TOF-MS to obtain the Peptide Mass Fingerprinting (PMF). The results showed that hGH-L1 matched a protein with NCBI accession number gi|156119322, which has an isoelectric point of 6.1 and was called “Low molecular 30 kDa lipoprotein PBMHPC-19 precursor (Figure 4b). hGH-L2 matched with a protein with NCBI accession number of gi|266439, which has an isoelectric point of 6.9 and was called “Low molecular mass 30 kDa lipoprotein 21G1” (Figure 4c). According to the identification criteria that the protein score CI% should be >95%, both hGH-L1 and hGH-L2 were identified, with scores of 100 and 99.99, respectively.

### 4 Homology analysis and tertiary structure prediction

**Figure 5:**
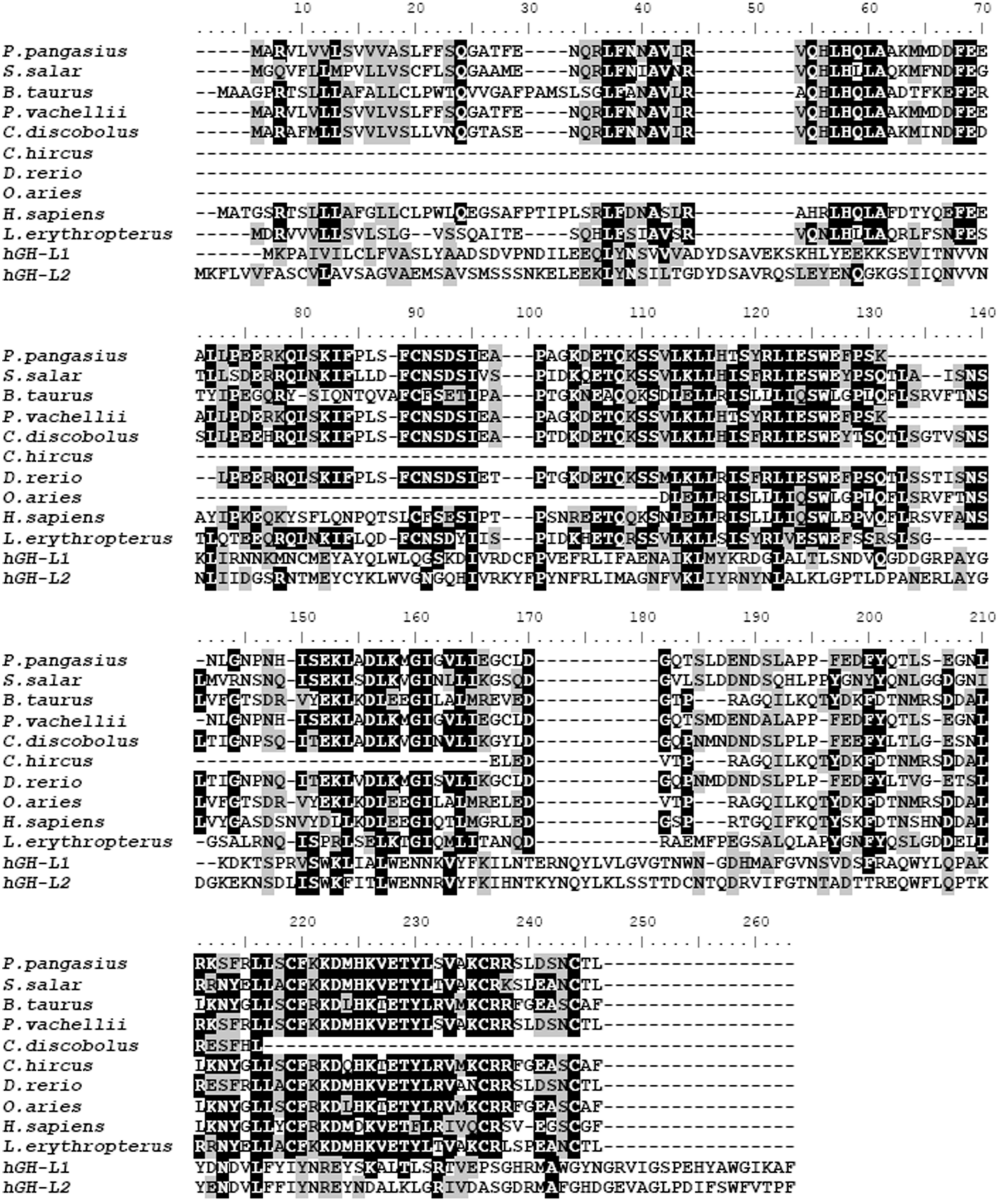
Multiple sequence alignment of BmhGH-like and GH proteins in different species.

**Figure 6:**
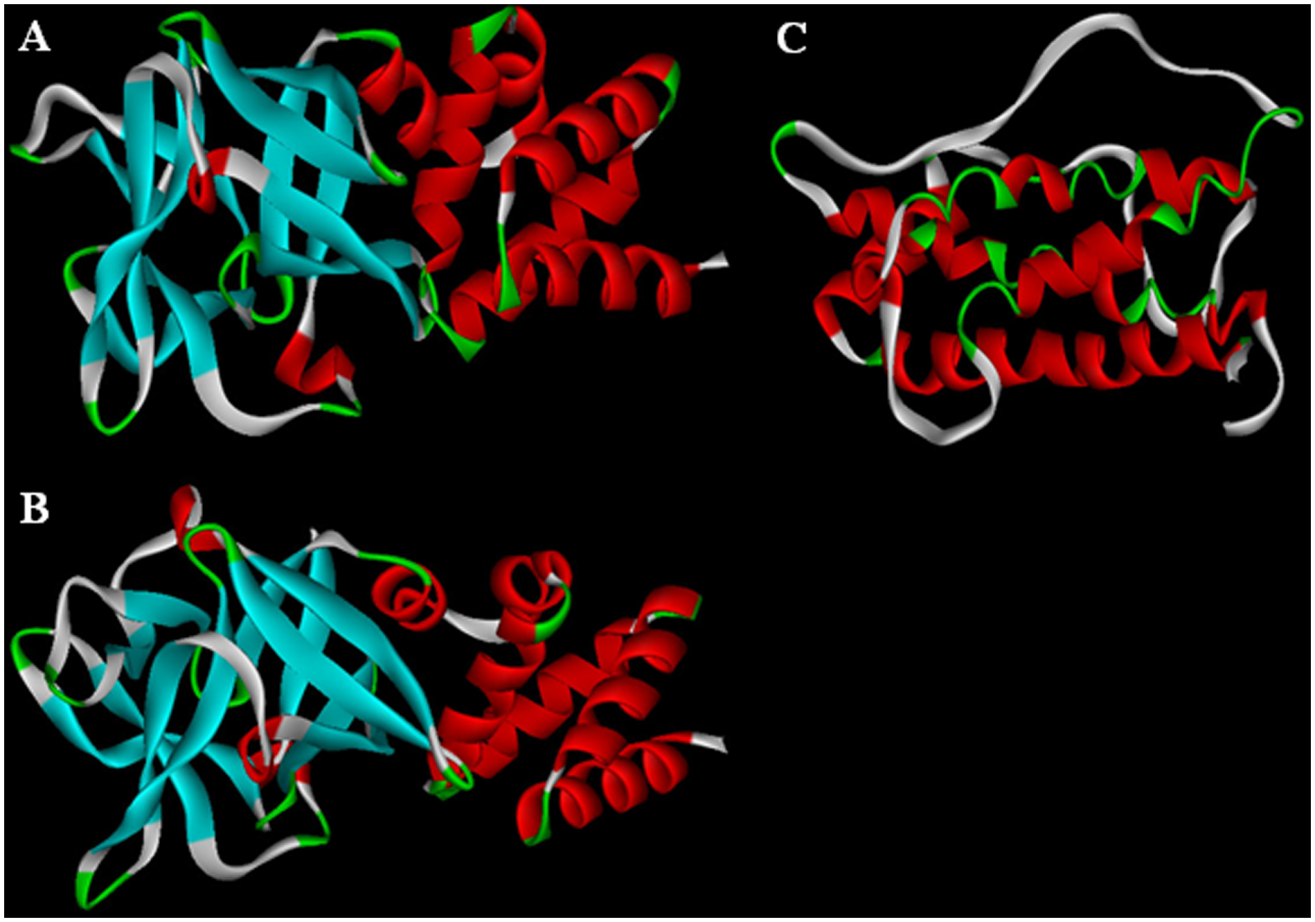
Comparison of three-dimensional structures of BmhGH-like proteins and hGH. A: hGH-L1; B: hGH-L2; C: hGH.

Analysis of conserved domains indicated that both hGH-L1 and hGH-L2 belonged to the β-trefoil superfamily of 30 kDa lipoprotein_11 [15], [16], and mainly composed of a signal peptide, α domain, and a β domain. hGH-L1 and hGH-L2 were shown to be homologous to GH protein sequences of different species (Figure 5). Further tertiary structure prediction found that the N termini of hGH-L1 and hGH-L2 had similar spatial structures to hGH proteins (Figure 6), which appear to be an α helix structure (Figure 6).

### 5 Investigation of biological functions of BmhGH-like protein

#### 5.1 In vitro measurement of growth-promoting effect of BmhGH-like protein

**Figure 7:**
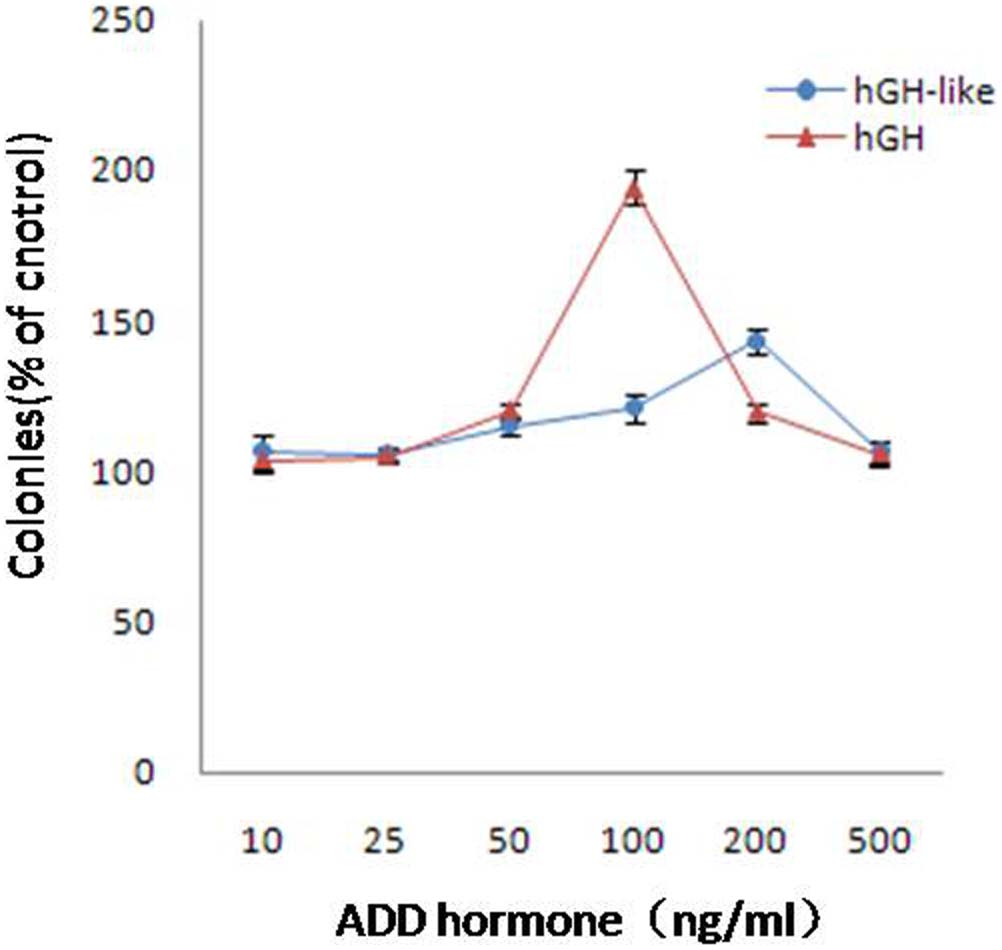
Effect of BmhGH-like protein on K562 colony formation.

Although K562 cells could not proliferate under serum-free culture conditions, the addition of hGH promoted proliferation, and the effect was remarkable even at 100 ng/ml. Meanwhile, the K562 cells also proliferated in the presence of 100 ng/ml purified BmhGH-like protein, and had the highest proliferation rate in the presence of 200 ng/ml protein (p<0.01). However, the proliferation-promoting effect of BmhGH-like protein was only half that of hGH. Cell proliferation was not detected at the concentration of 500 ng/ml (Figure 7).

#### 5.2 Effect of hGH-L2 protein on the cell cycle of BmN

**Figure 8:**
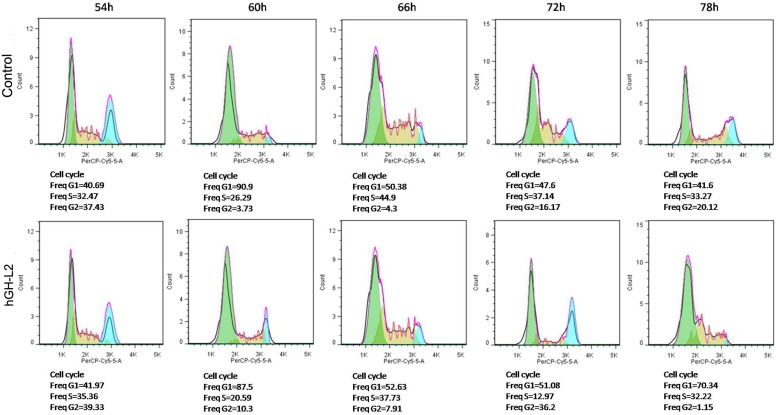
Cell cycle assay of BmN expressing hGH-L2.

To express BmhGH-like protein, recombinant Silkworm baculovirus expressing hGH-L2 were successfully prepared. BmN cells began to express the protein 60 h after infection. The flow cytometry analysis showed that compared with the cells infected with wild-type virus in the control group, the cells in the experimental group proliferated significantly faster 66 h after infection (p<0.05). During the infection, S phase cells decreased while G2 phase cells increased. Most cells entered M phase 72 h after infection. At 78 h after infection, the G0/G1 phase cells increased, and the cells divided and entered the next cell cycle. At this point, the cells in the control group were still in M phase (p<0.05) (Figure 8 shown).

## Discussion

Human growth hormone was discovered in the 1920s and was initially used to treat growth hormone deficiency. Further in-depth studies found that it is closely involved with immune function, circulatory system, urinary system, and aging, among others. [17], [18]. Recently, the clinical applications of growth hormone have been extended to the promotion of healing of wounds and burns, anti-infection and anti-inflammation, resistance to tissue and organ failure, treatment of fractures, provision of metabolic regulation and nutritional support for patients with severe multiple trauma, heart function improvement, immunity improvement, delaying senility, among other applications. However, the clinical use of hGH can also lead to side effects such as water sodium retention, intracranial hypertension, slipped epiphysis, and juvenile rheumatoid arthritis. Meanwhile, this is still controversial, the human body may produce antibodies against hormone drugs, thereby causing harmful side effects. Therefore, the exploration of hormone analogues for the reduction of side effects is one of the hotspots for studies on novel protein drugs.

In this study, an hGH-like protein that interacts with hGH antibodies was detected in the silkworm hemolymph and pupas for the first time [11]. This protein, called BmhGH-like protein, was purified from pupas and found to consist of two lipoproteins that belong to β-trefoil superfamily with molecular weights of approximately 30 kDa. The tertiary structures of the proteins included α domain and β domain, the latter of which is highly similar to the structure of hGH, and therefore might be the antigenic determinant that interacted with the hGH antibody. In vitro measurement of the biological activities using K562 cells found that BmhGH-like proteins could also promote K562 cell proliferation. To further confirm the growth promotion effect of BmhGH-like protein, recombinant BmNPV virus containing *hGH-L2* were used to infect silkworm oocyte BmN. We found that BmhGH-like protein shortened the G0/G1 phase, enabled cells to quickly enter S phase through the G1/S transition point and accelerate cell division, further demonstrating the growth hormone-like function of hGH-L protein.

Although the present work cannot reveal the mechanism of the growth promotion effect of the BmhGH-like protein on silkworm, the results confirm that these lipoproteins with similar structure and function to hGH are present in pupa, and that they can promote cell division and proliferation of BmN in silkworm and play important roles in the metamorphosis. These proteins have the potential to serve as a source of novel protein hormone drugs.

## References

[1] Lin Ling CG (2006) Progress in research on human growth hormone. Chinese Journal of Traditional and Western Medicine 17:1556–1558.

[2] Gauwerky C, Golde DW, Li CH (1980) Growth hormone polypeptides stimulate proliferation of K562 human erythroleukemia cells. J Clin Endocrinol Metab 51:1208–1210. 6448265 10.1210/jcem-51-5-1208

[3] Lechan RM, Nestler JL, Molitch ME (1981) Immunohistochemical identification of a novel substance with human growth hormone-like immunoreactivity in rat brain. Endocrinology 109:1950–1962. 7030717 10.1210/endo-109-6-1950

[4] Emmart EW, Wilhelmi AE (1968) Immunochemical studies with prolactin-like fractions of fish pituitaries. Gen Comp Endocrinol 11:515–527. 5726271 10.1016/0016-6480(68)90066-x

[5] Kaplan GW, Barrett RJ, Grayhack JT (1967) Extraction of a substance with prolactin-like and growth hormone-like properties from ovine testes: preliminary communication. J Urol 97:494–497. 4960047 10.1016/S0022-5347(17)63067-2

[6] Lechan RM, Nestler JL, Molitch ME (1981) Immunohistochemical identification of a novel substance with human growth hormone-like immunoreactivity in rat brain. Endocrinology 109:1950–1962. 7030717 10.1210/endo-109-6-1950

[7] Swinnen K, Broeck JV, Verhaert P, De Loof A (1990) Immunocytochemical localization of human growth hormone- and prolactin-like antigenic determinants in the insects, Locusta migratoria and Sarcophaga bullata. Comparative biochemistry and physiology. A, Comparative physiology 95:373–378. 1691964 10.1016/0300-9629(90)90235-k

[8] Ominato K, Nozaki M (2002) Distribution of growth hormone-like cells in the pituitary of adult sea lampreys, Petromyzon marinus. Zoolog Sci 19:1055–1059. 12362060 10.2108/zsj.19.1055

[9] Gallardo WG, Hagiwara A, Hara K, Soyano K (2006) Growth hormone-like substance in the rotifer Brachionus plicatilis. FISHERIES SCIENCE 72:781–786.

[10] Phares CK (1988) Use of receptor affinity chromatography in purification of the growth hormone-like factor produced by plerocercoids of the tapeworm Spirometra mansonoides. 8:645–665. 10.3109/10799898809049017 3392699

[11] Lan H, Nie Z, Liu Y, Lv Z, Liu Y, et al (2010) In vivo bioassay of recombinant human growth hormone synthesized in B. mori pupae. Journal of biomedicine & biotechnology 2010:306462. 20339512 10.1155/2010/306462 PMC2842897

[12] Peng H, Yuan X, Shi R, Wei X, Ren S, et al (2013) PHII-7 inhibits cell growth and induces apoptosis in leukemia cell line K562 as well as its MDR- counterpart K562/A02 through producing reactive oxygen species. Eur J Pharmacol 718:459–468. 23911883 10.1016/j.ejphar.2013.07.038

[13] Chen DH, Yan D (2000) One-step purification of recombinant human growth hormone by monoclonal antibody affinity chromatography. Chinese Journal of Immunology 16:651.

[14] Gauwerky C, Golde DW, Li CH (1980) Growth hormone polypeptides stimulate proliferation of K562 human erythroleukemia cells. J Clin Endocrinol Metab 51:1208–1210. 6448265 10.1210/jcem-51-5-1208

[15] Yang JP, Ma XX, He YX, Li WF, Kang Y, et al (2011) Crystal structure of the 30 K protein from the silkworm *Bombyx mori* reveals a new member of the beta-trefoil superfamily. J Struct Biol 175:97–103. 21514389 10.1016/j.jsb.2011.04.003

[16] Hou Y, Zou Y, Wang F, Gong J, Zhong X, et al (2010) Comparative analysis of proteome maps of silkworm hemolymph during different developmental stages. Proteome Sci 8:45. 20822545 10.1186/1477-5956-8-45 PMC2944163

[17] Somers W, Ultsch M, De Vos AM, Kossiakoff AA (1994) The X-ray structure of a growth hormone-prolactin receptor complex. Nature 372:478–481. 7984244 10.1038/372478a0

[18] Obermayr RP, Mayerhofer L, Knechtelsdorfer M, Tragl KH, Geyer G (2003) The reduced release of GH by GHRH in 8 subjects aged 65–69 years is augmented considerably by rivastigmine, a drug for Alzheimer’s disease. Gerontology 49:191–195. 12679611 10.1159/000069175

